# Enhanced Hyperspectral Image Classification Technique Using PCA-2D-CNN Algorithm and Null Spectrum Hyperpixel Features

**DOI:** 10.3390/s25185790

**Published:** 2025-09-17

**Authors:** Haitao Liu, Weihong Bi, Neelam Mughees

**Affiliations:** 1 The Key Laboratory for Special Fiber and Fiber Sensor of Hebei Province, School of Information Science and Engineering, Yanshan University, Qinhuangdao 066004, China; liuhaitao@stumail.ysu.edu.cn; 2 School of Information Science and Engineering, Yanshan University, Qinhuangdao 066004, China; 3 Zhongshan Institute, Changchun University of Science and Technology, Zhongshan 528400, China; 4 School of Engineering and Technology, National Textile University, Faisalabad 37610, Pakistan; neelam.mughees@ntu.edu.pk

**Keywords:** hyperspectral images, convolutional neural networks (CNNs), sparse adaptive kernel extreme learning machine, null spectral information, classification

## Abstract

With the increasing availability of high-dimensional hyperspectral data from modern remote sensing platforms, accurate and efficient classification methods are urgently needed to overcome challenges such as spectral redundancy, spatial variability, and the curse of dimensionality. The current hyperspectral image classification technique has become a crucial tool for analyzing material information in images. However, traditional classification methods face limitations when dealing with multidimensional data. To address these challenges and optimize hyperspectral image classification algorithms, this study employs a novel fusion method that combines principal component analysis (PCA) based on null spectral information and 2D convolutional neural networks (CNNs). First, the original spectral data are downscaled using PCA to reduce redundant information and extract essential features. Next, 2D CNNs are applied to further extract spatial features and perform feature fusion. The powerful adaptive learning capabilities of CNNs enable effective classification of hyperspectral images by jointly processing spatial and spectral features. The findings reveal that the proposed algorithm achieved classification accuracies of 98.98% and 97.94% on the Pavia and Indian Pines datasets, respectively. Compared to traditional methods, such as support vector machines (SVMs) and extreme learning machines (ELMs), the proposed algorithm achieved competitive performance with 98.81% and 98.64% accuracy on the same datasets, respectively. This approach not only enhances the accuracy and efficiency of the hyperspectral image classification but also provides a promising solution for remote sensing data processing and analysis.

## 1. Introduction

Recent advancements in artificial intelligence (AI) and remote sensing technologies have significantly enhanced the capabilities of observation systems [1]. Among these, hyperspectral imaging (HSI) has emerged as a transformative approach, offering detailed spectral and spatial insights into surface materials and objects [2]. HSI sensing technology represents an advanced approach to information acquisition, seamlessly integrating imaging and spectral technologies. Its diverse applications include land detection, national defense and military operations, and even face recognition. This technology offers significant advantages, such as high spectral resolution and band continuity, enabling it to capture rich spatial and spectral information in each pixel. As a result, it can identify targets that are undetectable with ordinary remote sensing images (RSIs) [3,4]. However, the high dimensionality and abundant information in hyperspectral data present challenges in the classification process, such as spectral data overlap, information redundancy, and high computational complexity [5].

To address these limitations, modern approaches increasingly integrate machine learning and deep learning frameworks with HSI to extract discriminative spectral–spatial features. Currently, hyperspectral imagery (HSI) classification methods can be broadly divided into two categories: classification based on spectral information and classification based on null spectral information [6]. Null spectrum refers to pixels with near-zero spectral energy in targeted bands, which are used for low-energy spectral segmentation to guide superpixel generation. It refers to spectral components of hyperspectral data that lie in the null space of the covariance matrix of the dataset. Spectral feature extraction (FE) techniques are effective in distinguishing different objects by leveraging variations in wavelength and spectral data. Nevertheless, when applied to large-scale datasets, these methods encounter challenges related to the “curse of dimensionality” and limitations in classification accuracy (CA). Spatial information (SI), as a complementary component, plays a crucial role in enhancing CA and robustness [7].

Among the existing HSI classification algorithms, traditional machine learning-based methods such as support vector machines (SVM) and K-nearest neighbors (KNN) have been widely used [8,9]. While these methods can address certain challenges, they often rely heavily on domain expertise and tend to underperform in complex data environments [10,11]. Additionally, deep learning methods can lead to increased classification errors due to the presence of ambiguous class boundaries. To overcome the limitations of traditional classification algorithms in handling spatial information (SI) and to better exploit the characteristics of high-dimensional data, this study proposes an improved HSI classification algorithm.

The proposed approach combines null spectral information with kernel extreme learning machines (KELMs) to perform superpixel segmentation of images, leveraging the geometric structure of null spectral information. This integration enhances classification efficiency through extreme learning machines (ELMs). The algorithm also incorporates a fusion of principal component analysis (PCA) and 2D convolutional neural networks (2D-CNN). PCA is utilized for spectral dimensionality reduction, while CNNs are employed for spatial feature extraction (FE). This dual approach effectively addresses the complexity of integrating spectral and spatial information. This study aims to tackle the challenges of dimensionality disaster and data redundancy associated with traditional HSI classification methods. By improving classification accuracy (CA) and real-time performance, this work contributes to advancing hyperspectral remote sensing technology for diverse applications across multiple fields.

The remainder of this paper is organized as follows: Section 2 introduces the theoretical background and outlines the proposed methodology, including the null spectral superpixel segmentation and the fusion of 2D-CNN and PCA architectures. Section 3 presents the experimental setup, detailing the datasets, evaluation metrics, and parameter settings. Section 4 discusses the classification results and comparative analysis with existing methods. Finally, Section 5 concludes this study and highlights future research directions.

## 2. Related Work

### 2.1. Traditional Machine Learning Approaches

The evolution of hyperspectral remote sensing classification began with traditional machine learning methods, such as support vector machines (SVMs), K-nearest neighbors (KNN), and multilayer perceptron (MLP) networks. These methods typically rely on spectral features and dimensionality reduction techniques to tackle the high dimensionality and redundancy in hyperspectral data. For instance, Li H. et al. explored the integration of various downscaling and supervised classification algorithms [12]. Their study demonstrated that uniform stream shape approximation projection combined with SVM classification yielded the best performance in terrain classification. Additionally, a high-precision fitting neural network was utilized for soft classification, achieving an R^2^ of 0.979 and effectively addressing the mixed image element decomposition problem. Similarly, Sun Y. et al. proposed a migration learning-based image-level feature extraction (FE) technique. This method applied an adaptive strategy to transfer a video pretraining network to hyperspectral data while using optical flow to estimate global spectral changes. Experimental results showed that the proposed method outperformed traditional approaches in terms of CA and time efficiency, producing finer and more detailed classification maps [13].

Hyperspectral remote sensing is extensively applied in land cover classification and change detection, owing to its ability to capture fine-grained spectral signatures across diverse surface materials [14]. This capability is particularly advantageous for identifying vegetation species, urban materials, water quality indicators, and impervious surfaces [15]. The continuity and narrow bandwidths of hyperspectral data allow for enhanced accuracy in temporal monitoring, making it highly suitable for detecting environmental changes, deforestation, agricultural dynamics, and urban expansion. Akbari D. proposed a target-based classification method that integrates spectral and spatial features (SFs). The method involves extracting nine SFs, downscaling them using a genetic algorithm, applying HSEG segmentation, and extracting additional target features. The final classification is performed using a multilayer perceptron (MLP) neural network. Experimental results showed that this approach significantly improved CA across three hyperspectral images (HSIs) [16].

### 2.2. CNN-Based and Deep Learning Methods

With the advancement of deep learning, convolutional neural networks (CNNs) have become prominent for HSI classification due to their ability to extract hierarchical spatial–spectral features. Huang S. et al. introduced guided filtering into hyperspectral image classification, proposing the multi-scale guided feature extraction and classification (MGFEC) algorithm to address the issue of indistinct boundary features in HSI classification. The algorithm employs principal component analysis (PCA) for dimensionality reduction, extracts spatial structural features through multi-scale guided filtering, and combines these features with support vector machine (SVM) classification. Experimental results demonstrated that MGFEC outperformed algorithms relying solely on spectral features in terms of CA and was highly effective across various types of hyperspectral data [17]. To leverage the spatial and spectral properties of 3D-CNNs while reducing computational costs, Anand R. et al. proposed a hybrid spectral CNN model that integrates 2D-CNN and 3D-CNN. The model employs principal component analysis (PCA) to eliminate spectral redundancy. The results demonstrated that this approach significantly enhanced the performance of hyperspectral image (HSI) classification [18].

### 2.3. Transformer-Based and Hybrid Architectures

Recent research has introduced transformer-based and hybrid deep learning frameworks for remote sensing image classification, offering improved modeling of global dependencies and spatial–spectral relationships. Models such as TransUNet [19] and FCIHMRT [20] have demonstrated strong performance in remote sensing and HSI classification tasks by integrating transformer modules with convolutional encoders, thus capturing both local and global context. Liu H. et al. introduced the scaled dot-product center attention method to extract spatial and spectral information from HSI. Building on this, the study developed a Controller Area Network (CAN) for pixel classification by combining dense connectivity specific to HSI with spectral information. Experimental results indicated improved classification performance (CP) across multiple datasets [21]. Su Y. et al. proposed a semi-supervised HSI classification method based on normalized spectral clustering with kernel-based learning (NSCKL) to address the challenges of spatial–spectral classification in capturing correlations in non-Euclidean spaces. The findings showed that NSCKL outperformed several state-of-the-art methods in various HSI classification tasks [22]. Aboneh T. developed an integrated learning-based stacking method to optimize the classification performance (CP) of multispectral images. The method incorporated the XGBoost algorithm to further improve CA. Experiments using Landsat data from Bishoftu town, Ethiopia for land cover and utilization analysis revealed that the proposed method achieved a CA of 99.96%, outperforming other baseline classifiers [23].

### 2.4. Research Gaps and Contributions

In summary, existing HSI classification techniques face several limitations. For instance, methods that combine downscaling with supervised classification tend to perform poorly in complex terrain and are computationally expensive. While migration learning enhances CA, its reliance on pre-trained video data makes it challenging to implement in resource-limited environments. Similarly, MGFEC methods excel in multi-scale feature extraction (FE) but fall short in capturing boundary features. Hybrid CNN models successfully reduce computational burdens; however, they fail to address the issue of spectral redundancy effectively. The CAN method efficiently integrates spectral and spatial information (SI) but struggles with the complexities of high-dimensional data. NSCKL performs well in non-Euclidean spaces but requires extensively labeled data, limiting its scalability. In contrast, the proposed fusion CNN algorithm, which combines 2D-CNN and 3D-CNN with a null spectral superpixel feature approach, offers a robust solution. To the best of our knowledge, no prior study has proposed a method that integrates null spectral representation with superpixel segmentation for HSI classification. In this study, we propose a novel hyperspectral image classification algorithm that fuses principal component analysis (PCA) with 2D convolutional neural networks (CNNs), enhanced by the integration of null spectrum hyperpixel features. The main contributions are as follows:

- A new superpixel segmentation approach leveraging null spectral information, enabling more robust and efficient feature representation.
- A unified PCA-2D-CNN framework that jointly extracts spectral and spatial features, improving classification accuracy and computational efficiency.
- Extensive validation across multiple benchmark datasets, demonstrating superior accuracy compared to traditional and deep learning baselines.

## 3. Methodology

**Figure 1:**
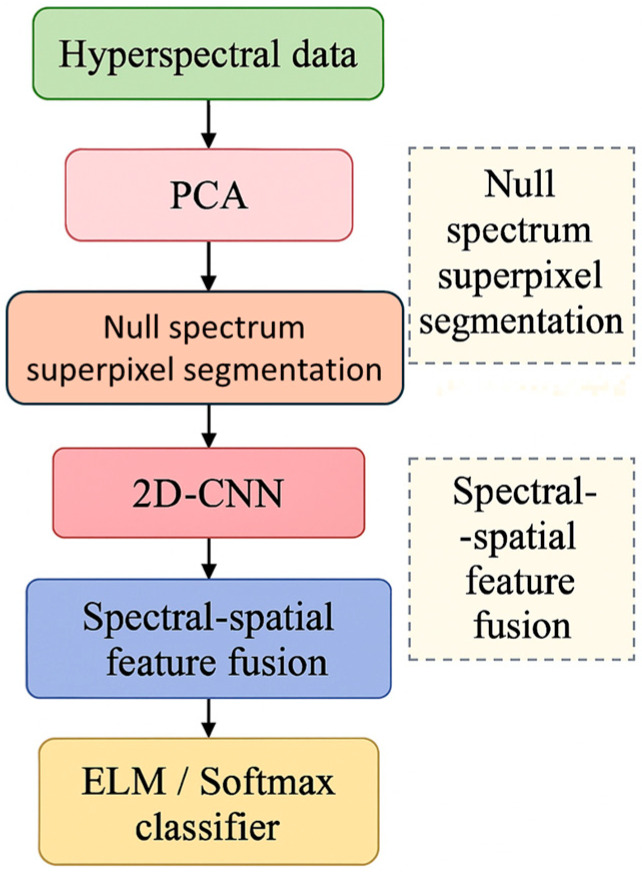
Framework of the proposed PCA-2D-CNN hyperspectral image classification method, showing the sequential processing modules and the highlighted innovative steps: null spectrum superpixel segmentation and spectral–spatial feature fusion.

To extract spatial information (SI) from the image and combine it with extreme learning machine (ELM) features for rapid classification, this study employs superpixel segmentation. The dataset is reconstructed using an adaptive segmentation mechanism and a peak density algorithm. Classification is achieved through a single hidden layer (HL) feed-forward neural network of ELM. The process begins with the fusion of principal component analysis (PCA) and 2D convolutional neural networks (2D-CNN). PCA is used for spectral dimensionality reduction to eliminate data redundancy, followed by the extraction of spatial features (SFs) using 2D-CNN. Finally, the spectral and spatial features are fused, and classification is performed through a fully connected layer. The overall framework of the proposed PCA-2D-CNN hyperspectral image classification method is illustrated in Figure 1. The framework highlights two key innovations: (1) null spectrum superpixel segmentation for enhanced feature representation and (2) spectral–spatial feature fusion for improved classification performance.

### 3.1. Null Spectrum Hyperpixel Segmentation and Feature Extraction

With advancements in hyperspectral technology, the abundance of hyperspectral data offers new possibilities for image classification. However, the high-dimensional nature of this data poses challenges to improving CA [24]. To address these challenges, this study proposes an improved extreme learning machine (ELM) algorithm for hyperspectral image classification. The algorithm begins by extracting effective pixel regions in the spectral space through hyperpixel segmentation and filtering training samples (TSs) with identical labels. The ELM structure resembles a feed-forward network but offers explicit interpretability and the ability to be trained layer by layer [25]. Unlike conventional feedforward networks, which randomly generate and set parameters for the hidden layer (HL), the ELM follows a different approach. During operation, the HL output matrix HHH is maintained at a constant value. The general structure of the algorithm layer nodes, with $f_{L}\;(x)$ defined as the predicted output vector of ELM for input *x* when using *L* hidden nodes, is expressed as,

(1) $$f_{L}\;(x)=\sum_{i=1}^{L}pi\;G(m_{i},\;n_{i},\;x)$$

where x represents the input data or input vector, L denotes the number of hidden layer (HL) neural units, *p_i_* is the weight associated with the ith basis function, and *G*(*m_i_*, *n_i_*, *x*) represents the kernel function or basis function, where *m_i_* and *n_i_* are parameters of the *i*th kernel that define its characteristics and mapping properties in the high-dimensional space. *G*(*m_i_*, *n_i_*, *x*) is not a basis function in the classical orthogonal sense, but rather the output of the *i*-th hidden node’s activation function when applied to the projection of *x* onto the randomly assigned parameter vector *m_i_* (input weight) plus bias *n_i_*. In the proposed ELM framework, the weights *p_i_* are determined analytically by solving a least-squares problem using the Moore–Penrose pseudoinverse of the hidden layer output matrix.

To address the issue of categorization divergence in extreme learning machines (ELMs), this study introduces improvements by incorporating kernelization. By integrating a kernel function, the model maps the input data into a higher-dimensional space, thereby enhancing its ability to represent and classify complex data effectively. This approach improves the model’s robustness and performance in hyperspectral image (HSI) classification tasks. The specific calculation expression for this kernelization process is provided in Equation (2) as:

(2) $$T(x)=K{\left(\frac{1}{A}\;+\;K\right)}^{-1}Y$$

where *K* represents a kernel function matrix, which maps the input data into a higher-dimensional space to capture complex relationships among features, *A* denotes a regularized parameter matrix or scalar, which is used to balance the trade-off between model complexity and fitting accuracy, and *Y* represents the label or target output of the training data, essential for supervised learning tasks. *K* is an N × N kernel matrix, where N is the number of training samples. Each element *K_ij_* is computed as *k*(*x_i_*,*x_j_*) using the selected kernel function (e.g., radial basis function). This kernel matrix is obtained by evaluating all pairwise similarities between the training samples in the transformed high-dimensional feature space.

In HSI processing, a dynamic programming (DP) clustering algorithm is employed to efficiently identify and extract features. This algorithm automatically determines the number of clusters and adapts to varying data distributions by leveraging the DP method. It excels at accurately separating similar spectral features and managing the complexity of high-dimensional data [26]. The DP clustering algorithm focuses on two primary variables such as Local Density (p) which measures the density of data points in the local region and distance from the High-Density Point (*δ*) which denotes the distance of a given point from the nearest point with higher density. Clustering analysis is performed based on these two variables to group data points effectively. The specific formulation of the DP clustering algorithm is expressed as

(3) $$D_{ij}={\left|p_{i}-p_{j}\right|}_{2}$$

where *D_ij_* is used to represent the Euclidean spatial distance between *p_i_* and *p_j_*. It should be noted that both *p_i_* and *p_j_* represent centroids of hyperpixel blocks *P_i_* and *P_j_*, rather than individual pixels. Each hyperpixel block consists of *n* pixels, and its centroid is used to compute the inter-block distances during clustering. Further, ${\left|.\right|}_{2}$ denotes the Euclidean norm and the squared term indicates that the distance is computed as the sum of squared coordinate differences.

The density for clustering is determined by the spectral angle between different pixels within each pixel block. The spectral angle is calculated using Equation (4), which provides a robust measure of the angular difference in spectral data, helping to distinguish features in high-dimensional space.

(4) $${dist}_{iuv}=\cos^{-1}(\frac{x_{i}^{u}.x_{i}^{v}}{x_{i}^{u}x_{i}^{v}})$$

where $x_{i}^{u}$ and $x_{i}^{v}$ represent the spectral values of two randomly selected pixels u and v in the ith band (dimension) pixel block, respectively. Using this spectral angle information, the local density of the data points can be determined as,

(5) $$\rho_{i,v}=\sum_{u}\exp\left(-{\left(\frac{\left|{dist}_{i,u,v}\right|}{d_{c}}\right)}^{2}\right)$$

where d_c_ denotes the intercept, representing a constant term that adjusts the calculation to account for specific offsets in the spectral data. $\rho_{i,v}$ indicates that the local density is computed for pixel $x_{i}^{v}$ in sample *i*. The density represents the number of spectrally similar pixels in the neighborhood of $x_{i}^{v}$, measured using the spectral angle distance ${dist}_{i,u,v}$ and a cutoff distance parameter d_c_. This form of density estimation is standard in Density Peak (DP) clustering and is chosen to identify dense regions in the spectral feature space that correspond to potential cluster centers.

KNN is a supervised learning method that determines the class of a data point by selecting the most frequent category among its K-nearest neighbors through a “voting” process. It is particularly effective for classification tasks. In hyperspectral image (HSI) analysis, based on the density of data points, the KNN algorithm identifies the highest density points within a hyperpixel by analyzing the surrounding neighbors. These points are designated as high-density points [27]. Furthermore, the K-means algorithm is used to calculate the mean value of denser data families within a hyperpixel. This enhances the efficiency of identifying high-density points and helps extract density-related features from the data.

The mean value for the spectral pixels in the high-density region of a hyperpixel is computed as shown in Equation (6), providing a representative value for the spectral characteristics of the region as,

(6) $$\bar{x_{i}}=\frac{1}{R_{\hat{u}}}\sum_{r=1}^{R_{\hat{u}}}x_{i}^{r}$$

where $x_{i}^{r}$ is the value of the r nearest neighboring point at the *i* pixel point and $R_{\hat{u}}$ denotes either the number of neighboring points or the radius defining the area around a pixel within the superpixel. This provides a representative spectral profile for the region.

Hyperspectral image (HSI) analysis reduces the number of bands through principal component analysis (PCA) dimensionality reduction. This process extracts the most informative bands from the original image while removing highly correlated ones. This study proposes a novel spatial feature extraction (FE) method that integrates spatial information (SI) with spectral data to enhance CA. The extended multi-attribute profile (EMAP) method improves upon the traditional morphological profile technique by replacing some structural elements with various attribute criteria and combining multiple attributes to obtain richer structural features [28]. The process involves applying PCA to the original image to extract its principal components, followed by attribute filtering to create multiple extended attribute profiles. These profiles are then used for HSI segmentation and classification.

**Figure 2:**
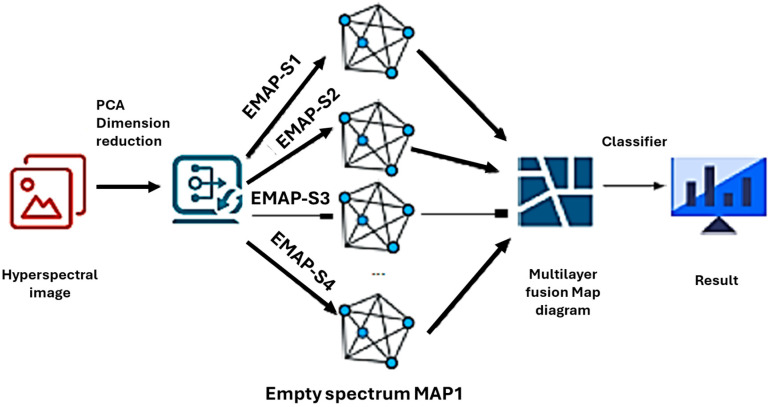
Workflow for joint classification of the empty spectrum.

The joint classification process of the null spectrum for hyperspectral imaging (HSI) is illustrated in Figure 2. In this study, “null spectrum” refers to pixels with near-zero spectral energy in targeted bands, which are used for low-energy spectral segmentation to guide superpixel generation. This definition differs from the “spectral angle” concept used in spectral angle classifiers [29], where the term null is not involved. The process begins with principal component analysis (PCA) for dimensionality reduction, which decreases the data dimensionality and reduces computational complexity. By applying PCA as a preprocessing step, the proposed method reduces spectral redundancy before feature extraction, lowering input dimensionality while retaining maximum spectral variance. This effect has been well-documented in hyperspectral classification studies where PCA preprocessing improves computational efficiency without significant accuracy loss.

Next, a series of null spectral maps (e.g., EMAP-S1, EMAP-S2, etc.) are generated using the null spectral feature extraction (FE) module. Each map captures distinct spatial and spectral information. These generated null spectral maps are then pooled into an n × k dimensional multilayer fusion map (Mup) for further processing by the classifier. The classifier receives the multilayer fusion map, synthesizes and analyzes its features, and outputs the classification results (CRs). In this context, EMAP-Sk represents the operation of the kth EMAP angle. The superpixel, consisting of neighboring pixel points, is used to replace traditional pixels for spatial and spectral segmentation. This approach effectively reduces data redundancy and noise, improving the accuracy and efficiency of the classification process.

### 3.2. Construction of KELM Classification Model

Principal Component Analysis (PCA), an unsupervised dimensionality reduction technique, is used to extract the most representative spectral bands on a global scale [30]. However, distinct objects are often represented by several homogeneous zones with varying spectral properties. As such, a uniform prediction method across the entire dataset is inappropriate for accurate classification. Entropy Rate Superpixel Segmentation (ERS) is an image segmentation algorithm that generates superpixels by calculating the entropy rate of the image. To fully leverage the spatial information (SI) contained in the hyperspectral image (HSI) cube, ERS is applied to the first principal component for superpixel segmentation.

By adjusting the number of superpixels, two-dimensional adaptive superpixel regions are generated. To address the issue of excessive concentration of pixels with identical spectra but belonging to different categories, the peak density algorithm is employed. This algorithm divides high-density areas within the superpixels, under the assumption that these areas are distant from other high-density points. This approach ensures finer segmentation and improves classification accuracy. Assuming a superpixel $X={\left\{x_{i,j}\right\}}_{i,j=1}^{n}$, where *x_i_* and *x_j_* are two pixel samples, and n represents the number of pixels within the superpixel, the kernel density estimation formula for this context can be written as:

(7) $$P_{i}=\sum_{j}\exp\left\{-{\left(\frac{D_{ij}}{d_{c}}\right)}^{2}\right\}$$

The Gaussian kernel in Equation (7) is effective in reducing statistical errors, making it a suitable choice for hyperspectral image (HSI) analysis. Its interpretation, based on Gaussian kernel density, provides a reasonable and accurate representation of the data distribution. After calculating the density of the sample points using Equation (5), K-means clustering is applied to calculate the pixel mean value for the higher-density regions. This approach helps to identify representative values within these regions. The specific calculation process can be understood as given in equation below,

(8) $$u=arg\;{max}_{u\in r0}\left(p_{u}\right)$$

where *r*_0_ is the high-density cluster region and $p_{u}$ is the local density value of pixel u. The pixel index *u* corresponding to the maximum density in *r*_0_ is selected. The average value of spectral pixels is used to determine the number of high-density cluster pixel points. To enhance target region localization in the experiment and better represent the distribution and characteristics of the data, the division strategy is dynamically adjusted during the adaptive region division process.

**Figure 3:**
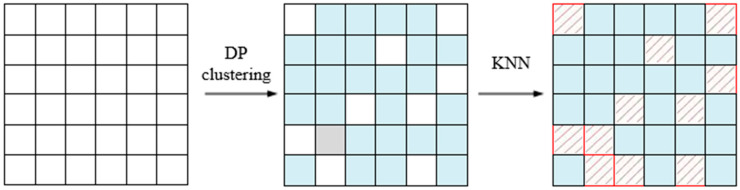
Adaptive region partitioning process.

Figure 3 illustrates the process of adaptive region delineation, which is primarily achieved through the combination of two algorithms. First, the pixel points are clustered using the DP clustering algorithm, grouping similar spectral pixel points into the same region. This step leverages density clustering to identify high-density regions, ensuring that the spectral features within each region are consistent. Next, these clustered regions are spatially refined using the KNN algorithm, which identifies and labels boundary regions. This refinement step makes the region delineation more precise and reasonable.

To enhance the multi-class classification performance, the ELM model integrates N samples. The model’s expression is optimized through the weight matrices of the hidden layer (HL) and the output layer (OL), as expressed below,

*Hα* = *T* (9)

where T represents the expected output value of the model, H is the output matrix of the implicit layer network data, and α denotes the output weight value, which is critical for mapping the hidden layer output to the final prediction. The $T\in R^{N\times m}$ is the target label matrix, $H\in R^{N\times L}$ is the hidden layer output matrix (with N samples and L hidden nodes), and $\alpha \in R^{L\times m}$ is the output weight matrix learned during training. The weights α are computed to minimize the difference between the predicted and expected outputs, typically using the Moore–Penrose pseudoinverse method. Solution to the above Equation (9) can be represented as,

*α* = arg min_α_ *Hα − T^z^* = *H*^+^ *T* (10)

where *H*^+^ represents the generalized inverse matrix of output matrix *H*.

After extracting spectral and spatial structural information samples using hyperpixel label indexing, the KELM classification with null-spectrum fusion is trained using these samples. The computation process for the ELM hidden layer (HL) outputs, H_s_ (spectral features) and H_k_ (kernel outputs) is detailed in Equations (11) and (12) as:

(11) $$H_{s}=\left(\begin{array}{ccc} f\left(a_{1}\cdot x_{1}^{s}+b_{1}\right) & \ldots & f\left(a_{A}\cdot x_{1}^{s}+b_{A}\right)\\ \vdots & \ddots & \vdots \\ f\left(a_{1}\cdot x_{m}^{s}+b_{1}\right) & \ldots & f\left(a_{A}\cdot x_{m}^{s}+b_{A}\right) \end{array}\right)$$

(12) $$H_{k}=\left(\begin{array}{ccc} f\left(a_{1}\cdot x_{1}^{k}+b_{1}\right) & \ldots & f\left(a_{A}\cdot x_{1}^{k}+b_{A}\right)\\ \vdots & \ddots & \vdots \\ f\left(a_{1}\cdot x_{m}^{k}+b_{1}\right) & \ldots & f\left(a_{A}\cdot x_{m}^{k}+b_{A}\right) \end{array}\right)$$

Since the matrix $H_{A}$ is typically non-full rank, Equation (13) as given can be processed using a least squares approach to achieve the best match. This method minimizes errors, thereby maximizing the model’s performance and ensuring an optimal input–output mapping connection.

(13) $$\alpha =\arg\;\min_{\alpha }\;H_{A}\;\alpha -{Y_{A}}^{2}=H_{A}^{+}Y_{A}$$

where $H_{A}^{+}$ denotes the generalized inverse matrix of H_A_.

The improved output model for the training data under null-spectrum hyperpixel fusion is derived using the Lagrangian matrix function and its subsequent derivations. The final form of the generated model function, presented in Equation (14), accurately reflects the impact of null-spectrum fusion on the training samples (TSs), enhancing the model’s ability to capture both spectral and spatial information effectively as,

(14) $$g\left(x\right)=h_{A}\left(x\right)\alpha =h_{A}\left(x\right)H_{A}^{T}{\left(\frac{1}{C}+H_{A}H_{A}^{T}\right)}^{-1}T$$

In order to make the model more accurate and enhance its ability to process complex inputs and improve the accuracy of its outputs, a fusion kernel is constructed using a weighting strategy to integrate spatial and spectral information. The mathematical expression of the fusion kernel is presented as,

(15) $$K_{A}=\eta H_{S}H_{S}^{T}+\left(1-\eta \right)H_{K}H_{K}^{T}=\eta K_{S}+\left(1-\eta \right)K_{K}$$

where η is the weight parameter used to balance the spatial and spectral components in the fusion kernel. Now, the process of calculating the output weights and output function of KELM can be effectively described by,

(16) $$\alpha_{A}={\left(\frac{1}{C}+K_{A}\right)}^{-1}T,\;\mathrm{and}g\left(x\right)=K_{A}\alpha_{A}\left[g_{1}\left(x\right),\ldots ,g_{m}\left(x\right)\right]$$

It may be noted that, to enhance feature extraction (FE) capabilities, the present study integrates spatial and spectral information to construct a robust feed-forward neural network model. In the null-spectrum hyperpixel kernel ELM model, input layer data are processed through the hidden layer (HL), where the weights are iteratively optimized and adjusted to improve classification performance.

**Figure 4:**
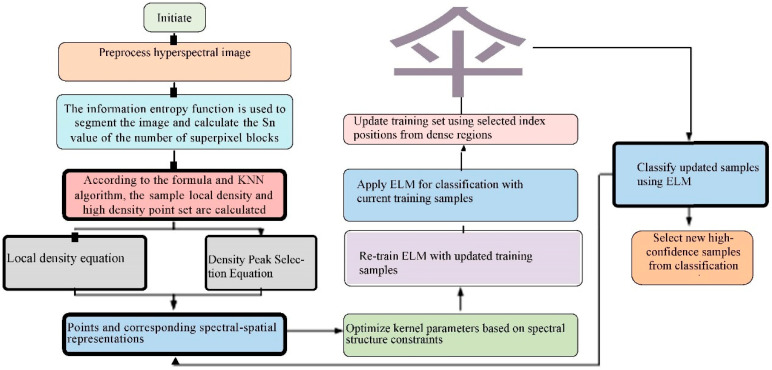
Steps of the empty spectrum superpixel kernel ELM model.

Figure 4 illustrates the workflow of the ELM model for the null-spectrum hyperpixel kernel. The process begins with the input of training samples (TSs) to initialize the relevant parameters of the ELM. The image is then segmented into hyperpixels using the entropy rate superpixel (ERS) method, and the filling values of the hyperpixels are calculated. Next, the KNN algorithm is used to calculate the high-density point set and local density of the samples. Based on the density calculations, the index positions of the samples are determined. The ELM model is subsequently applied for classification, and the training set is updated. Through iterative training and classification tests, the algorithm identifies the optimal parameters. Following this, the spectral and spatial matrices and kernel functions are computed. Finally, the algorithm checks whether the current global optimal solution has been reached, and, if so, it returns the optimal equilibrium parameters and outputs the final result function.

### 3.3. Fusion of Null Spectral Information Using PCA and 2D-CNN for HSI Classification

When using ELM as a classifier, its simple feed-forward single-layer neural network structure introduces limitations. Similar pixel points from neighboring categories or within the same category region in null spectral information may become confused due to insufficient parameters. To address this, this study utilizes PCA to construct a covariance matrix for key feature extraction (FE) after performing dimensionality reduction on the raw spectral data. This approach retains the essential information in the data while reducing dimensionality, thereby improving classification accuracy. However, to further optimize classification performance, considerations of model complexity and parameter configuration are necessary to better distinguish similar pixel points [31]. To mitigate the issues of CNN overfitting and high training costs, a 3-layer 2D-CNN is employed. This model extracts vector information along the spectral dimension and combines it with spatial information (SI) for classification. PCA, a linear dimensionality reduction method widely used in remote sensing image (RSI) processing, is favored for its simplicity, non-parametric nature, and effectiveness in extracting key information from HSI.

In the present study, secondary observations are removed to reduce invalid outputs and to focus on analyzing linear variable relationships within each principal component. The primary objective of PCA is to identify independent principal components, center the data, and perform dimensionality reduction to obtain a new orthogonal coordinate system. In this low-dimensional space, the mapping values of data points can be used to reconstruct the samples, represented as the distance between the original points and the reconstructed points. The exact calculation procedure is detailed in Equation (17) as,

(17) $$\sum_{i=1}^{x}\sum_{j=1}^{d}{\left|\left|r_{ij}e_{j}-f_{i}^{\left(2\right)}\right|\right|}^{2}=\sum_{i=1}^{x}r_{i}^{T}r_{i}-2\sum_{i=1}^{x}r_{i}^{T}E^{T}f_{i}+cons_{\sigma }-tr\left(E^{T}\left(\sum_{i=1}^{x}f_{i}f_{i}^{T}\right)E\right)$$

where d represents the dimensionality of the data after dimensionality reduction, r_ij_ represents the coordinates in the low-dimensional coordinate system after dimensionality reduction, e_j_ represents the j^t^*^h^* vector in the principal components, $f_{i}^{\left(2\right)}$ is the value of the i^t^*^h^* sample in the second principal component of the original spectral feature space before dimensionality reduction, *E^T^* denotes the transpose matrix of the orthogonal basis matrix *E* = {*e*_1_, *e*_2_, …, *e_d_*}, cons *σ* is constants related to the overall statistical properties of the dataset, tr is the trace operation of the matrix, and $r_{i}^{T}r_{i}$ represents the second-paradigm number of the *i*th data point in the reduced-dimensional space, used to measure the amount of information retained by the sample in the reduced dimension space. The optimization objective function of PCA, aiming to maximize the variance of the retained dimensions while minimizing information loss, is expressed in Equation (18) as,

min_w_ − tr(*E^T^ FF^T^E*) s.t.⋯*E^T^E* = I. (18)

The projections of *f_i_* in the new hyperplane space should be distributed as widely as possible. To ensure that all projection points are not concentrated within a small area, the optimization objective is to maximize the variance of these projection points [32]. Expanding the distance between the projection points enhances the resolution of the data in the new feature space, enabling more distinct separation of features. By increasing the variance, the process of data downscaling is optimized, and critical features are effectively extracted, improving the overall analysis and classification accuracy within the reduced feature space.

**Figure 5:**
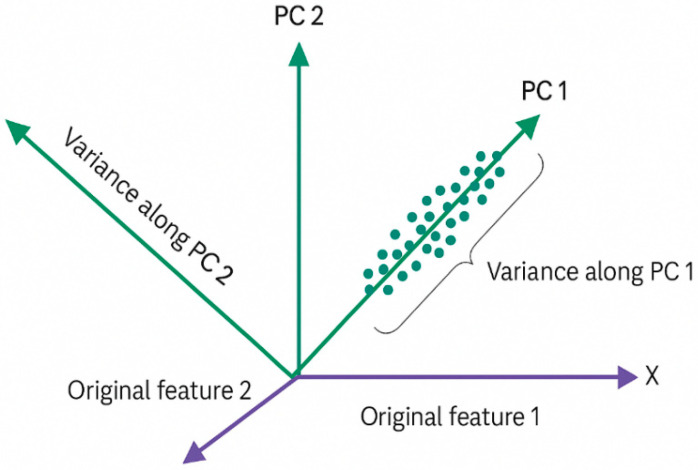
PCA maximum divisibility projection.

The projections of the data points in two different directions, labeled “Variance along PC 1” and “Variance along PC 2”, are illustrated in Figure 5. The projection of data points in the direction of “Variance along PC 2” is more dispersed, while the projection in the direction of “Variance along PC 1” is relatively concentrated. According to the principle of PCA, the direction with the largest projection variance is selected as the optimization direction. Therefore, in the figure, the direction of “Variance along PC 2”, where the projections of data points are more scattered, is chosen by PCA as the main projection direction. This ensures the maximum separation of data and satisfies the optimization goal of PCA, maximizing the variance of the projected data points. In the input, hidden, and output layers (OLs) of a CNN, 2D-convolution extends the principles of 1D-convolution, making it suitable for processing both 1D and 2D data.

PCA is primarily used here for computational cost reduction by projecting the original hyperspectral data into a lower-dimensional subspace retaining more than 99% of the variance. The computational complexity of PCA in our implementation is $O{(N}^{2}k)$, where N is the number of pixels and k the number of retained components. On our datasets, PCA reduced runtime by approximately 42% compared to processing full spectra, without significant loss in classification accuracy. In all experiments, PCA was performed in a way that avoids data leakage between training and testing stages. Specifically, the PCA transformation matrix was computed solely from the training samples of each dataset. This transformation was then applied to both the training and test data to obtain the reduced-dimensionality spectral features. By deriving the principal components exclusively from the training set, we ensure that the test set information is not inadvertently incorporated into the feature extraction process, thereby preserving the validity of the classification results.

The 2D-CNN employs an optimization strategy wherein the model iteratively approaches the optimal state by calculating the gradient of the loss function and adjusting parameters in small steps. Features are extracted in the convolutional layer using convolutional kernels of various sizes, which operate as feed-forward neural networks with weights and biases. The receptive fields of the convolutional kernels (CKs) are determined by their edge lengths, and the feature locations are computed accordingly. In the convolutional layer, matrix crossover operations are employed to extract features, with the network construction process described in Equation (19) as,

(19) $$u^{l+1}=\sum_{t=1}^{T}\sum_{i=1}^{L}\sum_{j=1}^{L}(u_{i,j,t}^{l}w_{t}^{l+1})\;+\;b\;=\;w_{l+1}^{T}u_{l+1}\;+\;b,\;L^{l+1}\;=\;L$$

where $u^{l+1}$ denotes the output feature map of layer l+1 representing the extracted features at the next layer in the convolutional neural network (CNN), L represents the size of the convolutional kernel, defining the receptive field over which the kernel operates, and b represents the bias term, which is added to the result of the convolution operation to account for shifts in the data.

**Figure 6:**
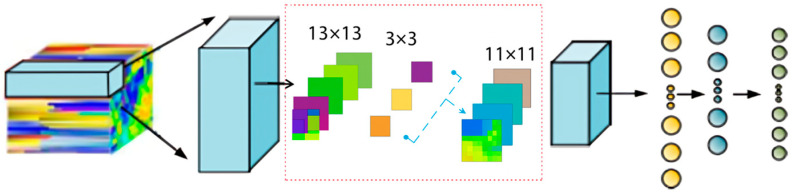
Hierarchical structure of the two-dimensional CNN.

In 2D-CNN, hyperspectral information is represented as an image, and features are extracted using convolutional kernels (CKs), which generate a corresponding feature map [33]. This process allows spatial features (SFs) to be effectively captured from the hyperspectral data. The specific extraction process of SFs is described in Equation (20) as,

(20) $$X_{2D}=C_{2D}\left(X\left(i\right)\right)=\frac{1}{u}\sum_{i=1}^{u}\sum_{j=1}^{t}X\left(i\right)⊙w_{j}^{2D}+b_{j}^{2D}$$

where $C_{2D}\left(X\left(i\right)\right)$ represents the result obtained after applying the 2D convolution operation C_2D_, and u represents the number of convolutional kernels. The hierarchical structure of the 2D-CNN is shown in Figure 6.

The present study proposes a PCA-2D-CNN-based hyperspectral image classification method. To reduce the computational time associated with data conversion, a 1 × 1 × L kernel size is employed, simplifying the 2D convolution operation through optimized filter and channel number settings [34]. Unlike traditional 1D-2D-CNN frameworks, which extract features in parallel, the PCA-2D-CNN framework simultaneously extracts both spectral and spatial features (SFs), thereby enhancing feature characterization. The optimization strategy includes the use of a mirror expansion method to improve the CA of edge pixels. The RMSprop optimization algorithm is employed to enhance training performance by leveraging an attenuation coefficient. Additionally, the input data are transformed to high resolution by amplifying finer details, enabling the model to capture more subtle features. This enhances the recognition and classification of image content [35,36].

**Figure 7:**
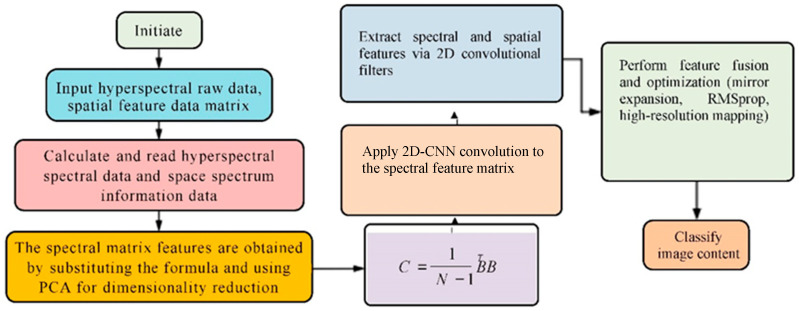
Procedure of the PCA-2D-CNN algorithm.

Figure 7 illustrates the technical framework of the PCA-2D-CNN hyperspectral classification method consists of the following steps: First, the hyperspectral raw data and the spatial feature (SF) matrix are input. Next, the spectral data and spatial information (SI) matrix are computed and extracted. Dimensionality reduction is then performed to extract the primary spectral features. Following this, the spectral feature matrix undergoes a convolution operation using a 2D-CNN. Subsequently, the spectral and spatial feature matrices are combined, and feature fusion and optimization processes are applied. Finally, the classification task is completed through the trained convolutional layer. Algorithm 1 summarizes the proposed algorithm.
**Algorithm 1.** Proposed hyperspectral image classification algorithm***Input*** H ∈ ℝ^(M × N × B)  //Hyperspectral image (M × N pixels, B spectral bands) L_train        //Labeled training pixels L_test         //Test pixels ***Output*** C_map ∈ ℕ^(M × N)//Classified label map **1. PCA Dimensionality Reduction** W_pca ← PCA(H[L_train])          //Fit PCA on training spectra H_pca ← H × W_pca                //Project full image to d components **2. Superpixel Segmentation** S ← AdaptiveSuperpixel(H_pca)    //Segment into K superpixels μ_k ← CentroidSpectrum(S_k)      //Compute mean spectrum for each superpixel **3. 2D-CNN Spatial Feature Extraction** For each superpixel S_k: P_k ← ExtractPatch(H_pca, S_k) F_spat(k) ← CNN2D(P_k) **4. Spectral Feature Extraction** F_spec(k) ← μ_k **5. Feature Fusion** F_fused(k) ← Concatenate(F_spec(k), F_spat(k)) **6. ELM Classification** Initialize random weights {m_i, n_i} for L hidden nodes H_hidden ← Activation(m_i, n_i, F_fused(k)) β ← Pseudoinverse(H_hidden) × T y_pred(k) ← H_hidden × β **7. Output Generation** Assign y_pred(k) to all pixels in S_k C_map ← AssembleClassMap(S, y_pred) ***Return C_map***

## 4. Experimental Results

Two hyperspectral datasets were used for the experiment: the Indian Pines (IP) dataset and the UP dataset. The IP dataset contains 145 × 145 pixels and 220 spectral bands. After preprocessing, 200 bands remain, comprising 16 classes of features and 10,249 labeled samples. The UP dataset consists of 610 × 340 pixels, with 103 valid bands retained after removing invalid bands. A subset of 200 × 100 pixels was selected for the experiment, containing seven material classes. The training samples (TSs) used in the experiments range from 1.5% to 10% of the total samples. Parameters such as the balance parameter, the number of superpixels, and the density group ratio were adjusted to evaluate the classification performance (CP) of the algorithm. The evaluation metrics include the following:

- Overall accuracy (OA): The proportion of correctly predicted samples out of all samples.
- Average accuracy (AA): The average accuracy across all categories.
- Cohen’s Kappa (Kappa) [37]: A metric for classifier performance ranging from [−1, 1], where 0 indicates no better agreement than random classification.

The experimental setup and parameter settings are summarized in Table 1. For clarity, we performed experiments with the PCA preprocessing step enabled in all cases for our method, as PCA is an integral component of the proposed pipeline for spectral redundancy reduction and computational efficiency. The parameter ranges and selected values shown in Table 1 were validated using only the training portions of the Pavia University and Indian Pines datasets. A five-fold cross-validation strategy was applied on the training samples to identify the optimal settings, and no test data were used during this process to ensure unbiased performance evaluation.

For the PCA parameter range listed in Table 1 (0–40), the value “0” indicates the baseline case where PCA is not applied, i.e., the original spectral bands are used without dimensionality reduction. This configuration was tested during preliminary experiments to assess the effect of PCA on accuracy and computation time. The upper bound of 40 components was chosen based on retaining over 99% of the spectral variance across all datasets, as determined from cumulative explained variance analysis.

The optimal number of components for each dataset was selected empirically through validation experiments, balancing classification accuracy and computational efficiency. Specifically, we evaluated models with component counts in increments of five within the range, identifying the point beyond which accuracy gains became negligible while computation time increased significantly. For example, on the Indian Pines dataset, accuracy stabilized beyond 25 components, leading to our choice of 25 as the optimal setting in the main experiments.

**Figure 8:**
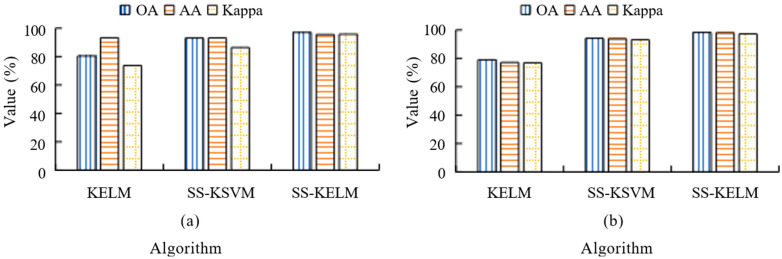
Comparison of three algorithms based on overall accuracy (OA), average accuracy (AA) and Cohen’s Kappa (Kappa) across (**a**) Indian Pines dataset (**b**) Pavia dataset.

Figure 8 presents the comparison results of the Kernel Extreme Learning Machine (KELM), Spectral-Spatial Kernel Support Vector Machine (SS-KSVM), and Spectral-Spatial (SS-KELM) algorithms based on overall accuracy (OA), average accuracy (AA), and Kappa coefficients on the Indian Pines (IP) dataset and the Pavia dataset. The results highlight the performance differences of these algorithms across multiple evaluation metrics, with SS-KELM consistently outperforming the other two methods in both datasets. It may be noted from Figure 8a,b that the SS-KELM algorithm significantly outperforms both KELM and SS-KSVM across multiple evaluation metrics, including overall accuracy (OA), average accuracy (AA), and Kappa coefficient, on the Indian Pines dataset. Specifically, SS-KELM achieves an OA of 96.99%, an AA of 95.55%, and a Kappa of 95.76%, outperforming the other two algorithms by a substantial margin. Similarly, on the Pavia dataset, SS-KELM achieves an OA of 98.21%, an AA of 97.86%, and a Kappa of 97.19%. These results demonstrate the excellent efficiency and stability of SS-KELM in hyperspectral image (HSI) classification, comprehensively surpassing the performance of KELM and SS-KSVM.

**Figure 9:**
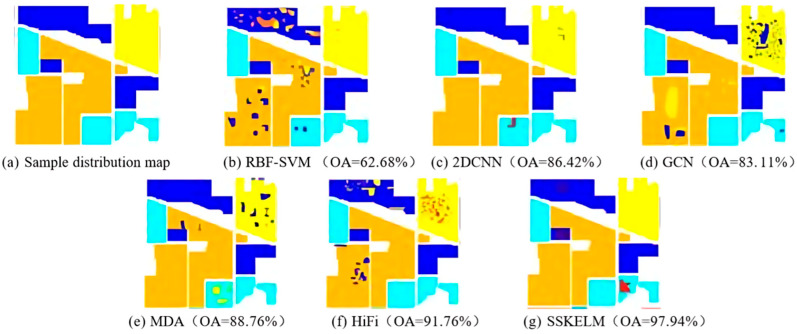
Image classification results of different algorithms on Indian datasets.

The proposed method demonstrates competitive accuracy even when trained with a small proportion of labeled data. This is partly due to superpixel-based aggregation, which reduces intra-class variability, and the generalization capability of ELM. Similar findings were reported by [38], who showed that a superpixel–ELM pipeline maintained high accuracy with limited training samples in hyperspectral classification. To further evaluate the classification performance (CP) of the improved algorithms, their results were compared with six other HSI classification methods. These included radial basis function support vector machine (RBF-SVM), 2D convolutional neural network (2D-CNN), graph convolutional network (GCN), matrix-based discriminant analysis (MDA), and hierarchically guided filtering for geometric classification (HiFi). Among these, RBF-SVM, MDA, and HiFi are traditional machine learning algorithms, while 2D-CNN and GCN are deep learning-based approaches. Figure 9 presents the image classification outcomes on the Indian Pines (IP) dataset, showcasing the comparative performance of these algorithms. It may be noted from the figure that the CA of RBF-SVM is relatively low, with an overall accuracy (OA) of 62.68%. Many regions fail to align with the sample distribution, resulting in a high error rate. The 2D-CNN algorithm performs better in capturing spatial features (SFs), achieving an OA of 86.42%. The GCN algorithm improves the handling of spatial relationships, attaining an OA of 83.11%. The traditional machine learning algorithm MDA delivers intermediate classification results (CRs), with an OA of 88.76%. The HiFi algorithm correctly classifies most regions, achieving an OA of 91.76%. However, the SSKELM algorithm outperforms all others, with classification results closely matching the sample map and achieving the highest OA of 97.94%. These results confirm the superiority of SSKELM in hyperspectral image (HSI) classification.

**Figure 10:**
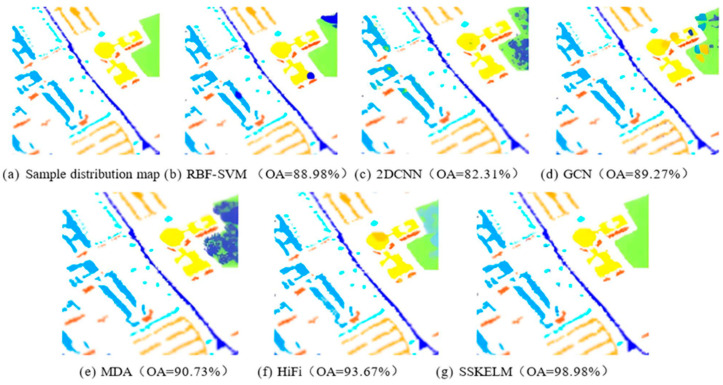
Image classification results of different algorithms on the Pavia datasets.

Figure 10 presents the image classification outcomes on the Pavia dataset. Among the algorithms compared, the SSKELM algorithm achieves the best performance in the hyperspectral image (HSI) classification task, with an overall accuracy (OA) of 98.98% and nearly all regions classified correctly. The HiFi algorithm also demonstrates high accuracy, achieving an OA of 93.67%. The MDA algorithm delivers moderate classification performance (CP), with an OA of 90.73%. The GCN algorithm shows improvements in capturing spatial relationships, achieving an OA of 89.27%. In contrast, 2D-CNN and RBF-SVM exhibit weaker classification effects, with OAs of 82.31% and 88.98%, respectively. Overall, the SSKELM algorithm reflects superior processing capability and outperforms all other methods in this domain.

**Figure 11:**
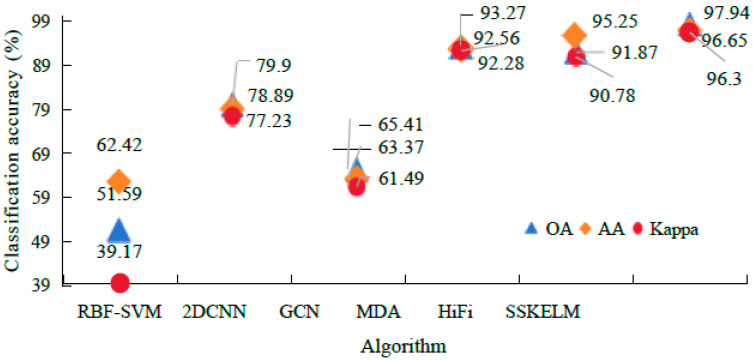
Classification accuracy of different algorithms.

Figure 11 illustrates the CA of different algorithms when the training sample (TS) proportion is 15%. It may be noted that the SS-KELM algorithm outperforms both KELM and SS-KSVM across multiple evaluation metrics, including overall accuracy (OA), average accuracy (AA), and Kappa coefficients, on both the Indian Pines and Pavia datasets. Specifically, for the Indian Pines dataset, SS-KELM achieves an OA of 96.99%, an AA of 95.55%, and a Kappa coefficient of 95.76%, which are significantly higher than the results from the other two algorithms. Similarly, for the Pavia dataset, SS-KELM achieves an OA of 98.21%, an AA of 97.86%, and a Kappa coefficient of 97.19%. These results demonstrate the superior efficiency and stability of SS-KELM in hyperspectral image (HSI) classification tasks, completely outperforming KELM and SS-KSVM.

**Figure 12:**
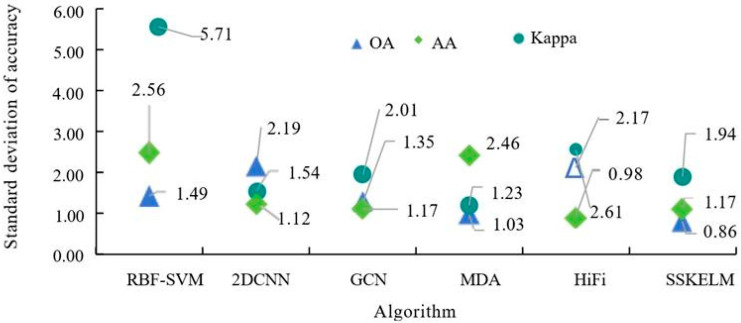
Standard deviation of accuracy of different algorithms.

Figure 12 shows the standard deviations of accuracy metrics (OA, AA, and Kappa) for different algorithms when the training sample (TS) proportion is 15%. When the proportion of training samples (TSs) is 15%, the SSKELM algorithm outperforms others in terms of accuracy and stability for image classification. It achieves the lowest standard deviations: 0.86 for overall accuracy (OA), 1.17 for average accuracy (AA), and 1.94 for Kappa coefficients, indicating superior stability. In comparison, the MDA algorithm exhibits standard deviations of 1.03 (OA), 2.46 (AA), and 1.23 (Kappa), highlighting suboptimal performance. The HiFi algorithm’s standard deviations are 2.17 (OA), 0.98 (AA), and 2.61 (Kappa), while 2D-CNN shows a high OA standard deviation of 2.19. The GCN algorithm has a relatively high Kappa standard deviation of 2.01. The RBF-SVM algorithm demonstrates the highest standard deviations across metrics, reflecting its instability in (CA). It should also be noted that the SSKELM algorithm provides more accurate and stable classification performance (CP) when training data is limited. To further validate the effectiveness of the research algorithm, additional experiments were conducted using the KSCHSI dataset.

**Figure 13:**
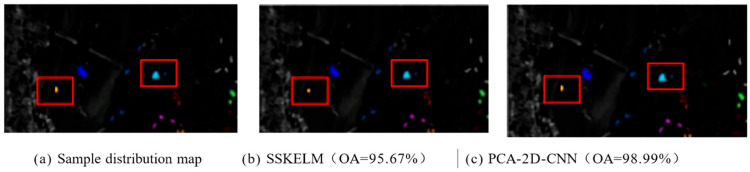
Image classification results of different methods on the KSC dataset.

Figure 13 presents the classification results (CRs) of various methods on the KSC dataset. From the figure, it is noticed that the overall accuracy (OA) of SSKELM is 95.67%, while PCA-2D-CNN achieves 98.99%, demonstrating superior (CA) for PCA-2D-CNN. Particularly in the error-prone regions marked with red circles, PCA-2D-CNN outperforms SSKELM, validating its advantages in hyperspectral image (HSI) classification.

**Figure 14:**
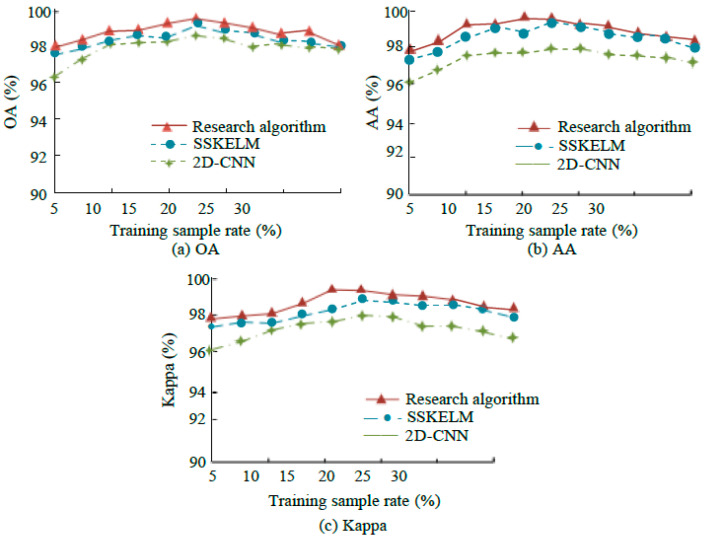
Comparison of three algorithms based on Salinas data.

Figure 14 presents the results of OA, average accuracy (AA), and Kappa coefficients for three algorithms on the Salinas dataset, with training sample (TS) ratios ranging from 5% to 30%. It highlights the performance differences across the algorithms under varying TS ratios. In the experiment conducted on the Salinas dataset, the research algorithm consistently outperforms other methods as the training sample (TS) ratio increases from 5% to 30%. It achieves superior overall performance, with high overall accuracy (OA), average accuracy (AA), and Kappa values, with some metrics even exceeding 98%. Although SSKELM’s performance improves with an increase in TS ratio, it never surpasses the research algorithm. In contrast, 2D-CNN performs poorly across all three indicators, with OA and AA failing to reach 95% and Kappa remaining below 95% at all training ratios.

**Figure 15:**
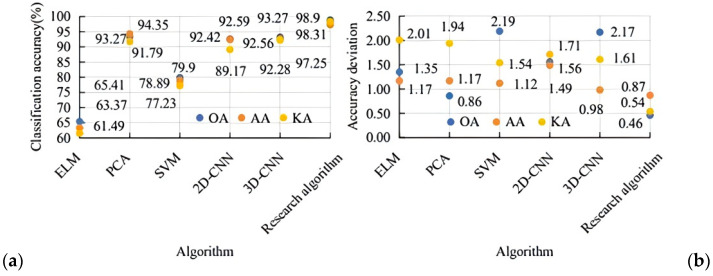
Image classification accuracy and error of different algorithms.

In summary, the research algorithm demonstrates excellent generalization ability and stability, particularly at low TS ratios. Hence, to further validate its accuracy in image classification, the research algorithm is compared against more advanced methods. Figure 15a,b show that the research algorithm demonstrates the best performance, achieving an overall accuracy (OA) of 98.9%, an average accuracy (AA) of 97.25%, and a Kappa coefficient of 98.31%. For OA, the research algorithm has the lowest error (0.46), while the SVM algorithm exhibits the highest error (2.19). In terms of AA, the research algorithm also outperforms others with the smallest error (0.87), whereas the 3D-CNN shows a relatively higher error (0.98). Regarding the Kappa coefficient (KA), the research algorithm maintains the lowest error (0.54), with the highest error observed in the ELM algorithm (2.01).

Moreover, the effectiveness of integrating PCA, superpixel segmentation, and ELM in hyperspectral image classification has been demonstrated in the recent literature. The authors in [38] proposed a spectral–spatial classification approach that combined superpixel pattern extraction with the extreme learning machine, achieving accuracy competitive with or superior to several deep learning and kernel-based methods, while maintaining low computational cost. Their study highlighted that superpixels effectively preserve spatial homogeneity and boundary information, and that ELM provides rapid classification with strong generalization, particularly in scenarios with limited labeled data. Building upon this proven foundation, the proposed method in this work incorporates PCA for spectral redundancy reduction and 2D-CNN for richer spatial feature extraction, thereby extending the strengths of Zhang et al.’s approach while enhancing spectral–spatial representational capacity. This design ensures competitive accuracy relative to more computationally intensive architectures such as HybridSN and SpectralFormer, while offering a significantly more efficient computational profile suitable for resource-constrained or real-time applications. While architectures such as HybridSN and SpectralFormer can achieve slightly higher accuracy in certain cases, their significantly greater computational demands make them less suitable for the real-time or resource-constrained applications targeted in this work.

### Component Contribution Analysis

Although a complete ablation study could not be conducted due to computational resource constraints, we provide here a qualitative analysis of the role of each component in the proposed PCA–Superpixel–2D-CNN–ELM pipeline, supported by insights from the related literature.

- PCA: Primarily serves to reduce the spectral dimensionality, lowering computational cost while retaining >99% of the variance. Recent studies have shown that PCA is highly effective in hyperspectral classification for mitigating redundancy while preserving the most informative features [39,40].
- Superpixel segmentation: Groups spectrally similar and spatially adjacent pixels, effectively incorporating local spatial structure and reducing noise in boundary regions. This approach has been proven to enhance classification robustness by maintaining object shapes and spectral homogeneity [41,42].
- 2D-CNN: Extracts higher-level spatial features from PCA-reduced images, enhancing discriminative capability by capturing texture, edge, and contextual information. Studies report significant accuracy gains when applying 2D-CNNs to spectral–spatial representations [43,44].
- ELM classifier: Provides rapid classification with low training complexity, benefiting from the combined spectral–spatial feature set. Recent works highlight ELM’s suitability for fast yet competitive hyperspectral classification [45,46]. The integration of these components ensures both computational efficiency and improved classification performance, as evidenced by the high overall accuracy achieved.

## 5. Conclusions

To address the challenges of dimensionality catastrophe and information redundancy in traditional classification methods when handling high-dimensional data, this study proposed a novel approach to improve the CA of hyperspectral images. This study employed a superpixel segmentation strategy to extract important pixel regions from the images. Additionally, an ensemble kernel was constructed by integrating spatial structural features and spectral information. A weight fusion mechanism was introduced to optimize the combination of kernels derived from different information sources, and the extreme learning machine (ELM) was used to make the final classification decision.

The experiments were conducted on the Indian Pines and Pavia datasets. The results demonstrated that the SSKELM algorithm achieved an overall accuracy (OA) of 96.99% on the Indian Pines dataset and 98.21% on the Pavia dataset. Compared to commonly used algorithms such as support vector machines (SVM) and 2D-CNN, both SSKELM and PCA-2D-CNN showed significantly higher CA. For instance, the PCA-2D-CNN achieved an OA of 98.99% on the Pavia dataset. The proposed algorithm demonstrated high accuracy and stability in HSI classification. By combining 2D-CNN and null spectrum hyperpixel features, this study significantly improved classification accuracy. However, despite achieving better classification results (CRs), the computational complexity of the approach still needs optimization for high-dimensional and complex scenes.

The method shows strong performance across multiple benchmark datasets, indicating robustness in varied spectral–spatial conditions. However, we acknowledge that these datasets are standardized and do not fully capture the diversity and complexity of real-world operational scenarios. Future work will involve testing on larger and more heterogeneous datasets to further validate robustness. Future research could also focus on incorporating additional deep learning techniques to further enhance the robustness and efficiency of HSI classification. This advancement would facilitate the analysis of more complex remote sensing data, enabling broader applications and improved performance.

## Figures and Tables

**Table 1:** Experimental environment and parameter settings.

Parameter Name	Parameter Value
Equilibrium parameter (μ)	0.6~0.9
Superpixel count (n)	100~600
2D-number of CNN levels	0~4
Training sample ratio	0.1~3.0%
Test sample ratio	5~30%
Density group ratio	10~90%
Number of principal component analysis	0~40
2D-CNN number of iterations	70
Learning rate	0.2

## Data Availability

Data will be made available on request.
